# Knowledge of breast cancer and breast self-examination practices and its barriers among university female students in Bangladesh: Findings from a cross-sectional study

**DOI:** 10.1371/journal.pone.0270417

**Published:** 2022-06-28

**Authors:** Rumpa Sarker, Md. Saiful Islam, Mst. Sabrina Moonajilin, Mahmudur Rahman, Hailay Abrha Gesesew, Paul R. Ward

**Affiliations:** 1 Department of Public Health and Informatics, Jahangirnagar University, Savar, Dhaka, Bangladesh; 2 Centre for Advanced Research Excellence in Public Health, Savar, Dhaka, Bangladesh; 3 Department of Epidemiology, School of Health Sciences, Mekelle University, Mekelle, Ethiopia; 4 Centre for Research on Health Policy, Torrens University Australia, Adelaide, Australia; Management and Science University, MALAYSIA

## Abstract

Early diagnosis of breast cancer is the best approach towards its control that may result in alleviating related mortality and morbidity. This study aimed to evaluate knowledge about breast cancer and both practices and perceived barriers to breast self-examination among female university students in Bangladesh. A cross-sectional study was carried out with 400 female students of Jahangirnagar University, Bangladesh. Participants were sampled from female dormitories at the university from January to April 2020. Proportionate stratified random sampling was conducted to calculate the study sample from each dormitory. A validated semi-structured self-reported questionnaire was employed to collect data from participants during the survey periods. The questionnaire consisted of demographic variables, items about knowledge about breast cancer, breast self-examination practices and its barriers. We applied descriptive and inferential statistics and data were analyzed using the Statistical Package for the Social Sciences (SPSS). Participants were aged between 18–26 years and comprised university students of first year (20%), second year (24%), third year (22%), fourth year (21%) and Master’s (14%). 18% of them reported positive family history (mother, aunt, sister/cousin, grandmother) of breast cancer. The overall mean score of total knowledge items was 15 (SD = 3) out of 43, with an overall correct rate of 34%. The mean score of total knowledge items was significantly higher (*p*<0.001) among Master’s students and students with family members who have had breast cancer. Only one in five students (21%) ever practiced breast self-examination. The mean score of practice of breast self-examination was significantly higher (*p*<0.001) among participants who reported having family member of breast cancer. Total knowledge score about breast cancer and practice of breast self-examination were significantly correlated with each other (*r* = 0.54; *p*<0.001). About 33% participants reported ‘lack of knowledge’ as the main barrier to practicing breast self-examination followed by ‘I do not have the symptoms’ (22%), and ‘shyness/ uncomfortable feelings’ (17%). The study revealed low levels of knowledge about breast cancer and low breast self-examination practices. Our findings highlight the need to develop, implement and promote socially, culturally and demographically appropriate educational interventions programs aimed at breast cancer and breast self-examination awareness and practice in Bangladesh.

## Introduction

Breast cancer is a major global health concern and a prominent reason of mortality among females. It is the most frequent cancer among women globally, impacting 2.1 million women each year, and is predicted to grow to approximately 3.2 million new cases per year by 2050 [1]. There were 13,028 new breast cancer cases (19% of all cancer types among females) in Bangladesh in 2020 [2]. Although the incidence rate is higher among women older than 50 years, the rate of diagnosis with breast cancer among young women under 50 years of age has increased. Breast cancer also causes the greatest number of cancer-related deaths among women [3]. For example, an estimated 627,000 women died from breast cancer in 2018 in Bangladesh, contributing to nearly 15% of all cancer deaths among women [4]. Delay in seeking treatment after diagnosis or seeking medical help after experiencing symptoms of breast cancer lowers the level of successful treatment outcome and thus decreased survival length [5]. But most breast cancer patients are diagnosed in developing countries, including Bangladesh, at an advanced stage due to a lack of understanding and insufficient access to health care facilities [6]. In Bangladesh, breast cancer is ranked as the 2^nd^ leading cancer after cervical cancer and together breast and cervical cancer account for 38% of all cancer among women. Generally, this disease occurs more frequently among older women above 50, but in recent years, the rising population in this age range has led to a surge in the absolute number of younger women diagnosed with breast cancer. According to Ahmadian and Samah (2012), Asian women get breast cancer at a younger age (40 to 49 years old), than their Western counterparts, who develop it at a later age, (between 50 to 59 years old) [7].

In Bangladesh, Mammography and MRI screening are available at some tertiary level hospitals. However, mammography is more sensitive at detecting cancer in older women [8] and MRI is expensive and it may detect more changes that require investigation but turn out to be non-cancerous (about one in ten chance of this) and it has not been shown to improve overall survival in women who are screened. It is currently only considered for young women at very high risk [9]. For breast cancer screening, Bangladesh has adopted the clinical breast examination (CBE) method a simple and low-cost method, feasible in low resource settings that allows rapid training for the providers. Currently, 271 Upazila Health Complexes (UHCs) have established CBE centers in all districts of Bangladesh and the current training is equipping senior staff nurses from 14 additional UHCs located in 7 Districts to conduct screening [10].

The American Cancer Society also recommends that women should be familiar with how their breasts normally feel via breast self-examination (BSE) and report any breast changes promptly to their health care providers. The ‘National Cancer Control Strategy and Plan of Action 2009–15’ in Bangladesh advocates encouraging clinical breast examination (CBE) and BSE as breast cancer’s early detection approach for disease downstage and survival improvement [11]. In addition, the Breast Health Global Initiative (BHGI) guideline for low- and middle-income countries suggests BSE as the first step in preventing breast cancer [12].

BSE is an easy, expedient, non-invasive and no-cost way to check out women’s own breasts to find any changes in their breasts. BSE can identify symptoms of breast cancer at early stages of cancer, when the condition can be more successfully treated and thus increasing survival rate from breast cancer. BSE aids women by making them conversant about how their breasts should look and feel thus leading to ‘breast awareness’ and also enable them to identify changes in their breasts in the initial stage [13]. It can be performed on a regular basis, at any age and is suitable for low resource countries like Bangladesh. Conversely, mammography screening is not a practical approach to pursue breast cancer prevention due to its high costs for the health system and individual women (in terms of out-of-pocket costs). Although inappropriate or inaccurate BSE enactment may produce both false positives and false negatives for women, BSE is still regarded as a legitimate and realistic alternative for early breast cancer screening in women [14].

In Bangladesh, a previous study reported poor knowledge and awareness of breast cancer and lack of adherence to any recommended screening method of breast cancer, including BSE [15]. However, very few studies have been conducted with young and educated females who are within their reproductive age (i.e. female university students). It is crucial to assess their level of knowledge and practice of BSE as this assessment of knowledge may reflect the awareness level of a large proportion of the population. Although breast cancer incidence is lower in this age group of women than for older women, it is important for younger women to practice BSE in order to identify changes in breast tissue in the future and hopefully prevent incurable, late-stage cancers. In addition, our sample includes more educated young women, and may not reflect knowledge, attitudes and practices of BSE with less educated young women in Bangladesh (different studies may be required with these young women). Nevertheless, using the case of Jahangirnagar University, this study aimed to investigate the knowledge of symptoms, risk factors, treatment modalities and screening methods of breast cancer among young females who represent the most educated segment of population, as well as to examine the practice of BSE and the barriers that are hindering the practice of BSE.

## Methodology

### Participants and procedures

A cross-sectional study was carried out with 400 female students who enrolled in Bachelor and Master’s programs of Jahangirnagar University (fully residential university of Bangladesh). The study was solely conducted in the 8 female dormitories and data were collected over four months from January-April 2020. At first, for sample size determination, Yamane’s simplified sampling formula was used, which calculated our sample size required as 386 participants. However, in order to be even more confident, we decided to slightly over-sample, and aim for 400 participants. We then applied proportionate stratified random sampling, based on the population proportion of dormitories in order to calculate the study sample from each dormitory. Using this approach, each stratum sample size is directly proportional to the population size of the entire population of strata.

The inclusion criteria included: (ⅰ) being 18 years of above; (ⅱ) being female university students who reside at dormitories; and (ⅲ) being willing to take part in the survey. The exclusion criteria were being below 18 years old and having incomplete responses.

### Study instruments

A pre-tested, self-reported, semi-structured questionnaire including informed consent, socio-demographic information and questions related to knowledge of breast cancer, BSE practices and its barriers, was prepared for the study through extensive literature review [16–20]. In order to add further face validity, the questionnaire was reviewed by an external reviewer who is an oncologist with extensive experience of consulting women in Bangladesh about breast cancer prevention, diagnosis and prognosis. Likewise, a pilot test was conducted to assess the readability of the questionnaire. The questionnaire was finalized after incorporating minor amendments based on the participants’ feedback during the piloting periods. The survey questionnaire is attached in the **S1 File**.

#### Socio-demographic information

Socio-demographic information was recorded during the survey including age, study year (first/ second/ third/ fourth/ Master’s), marital status (unmarried/ unmarried), family history of breast cancer (yes/ no), and relationship with breast cancer affected patient (mother/ sister/ cousin/ aunt/ grandmother).

#### Knowledge of breast cancer measures

To assess the participants’ knowledge of breast cancer, a total of 43 questions regarding breast cancer (i.e., 8 for symptoms, 10 for risk factors, 6 for treatment, 8 for prevention, 5 for screening, and 5 for process of BSE) were asked during the survey. Each question has three possible responses (i.e., yes/no/don’t know). The distributions (frequencies and percentages) of all questions are presented in **S2 File**.

#### BSE practices and its barriers measures

A single construct (i.e., *Have you ever self-examined your breast for breast cancer*?) was used to assess the BSE with binary responses (yes/no). In addition, the barriers of BSE were also recorded.

### Data analysis

Data analysis was performed using the SPSS version 25. Descriptive statistics such as frequencies and percentages were computed for categorical variables; whereas, means and standard deviations were for continuous variables. Some first-order analyses (i.e. t-tests and one-way ANOVA) were performed to assess the association between independent and dependent variables. The Pearson correlation test was also carried out to find out the correlations between two continuous variables. The significance level (*p*-value) was set at 0.05.

### Ethical considerations

The study protocol was reviewed and approved by the Biosafety, Biosecurity, and Ethical Clearance Committee, Jahangirnagar University, Savar, Dhaka-1342, Bangladesh. Before starting data collection, participants were informed about the objectives, methodology and the approximate time to complete the survey. Then written informed consent was obtained from each participant. Likewise, participants were also assured that all information related to them will be kept confidential and anonymous.

## Results

A total of 400 female participants aged between 18–26 years were included in the final analysis. The participants comprised of university students of first year (19.5%), second year (23.5%), third year (21.8%), fourth year (21.0%) and Master’s (14.2%) (Table 1). Most of them were unmarried (86.0%). A sizable minority reported they had family history of breast cancer (18.3%). The participants also reported the relationship with affected people as follows: mother (11.6%; n = 8), sister/cousin (24.6%; n = 17), aunt (40.6%; n = 28) and grandmother (23.2%; n = 16).

**Table 1:** Characteristics of participants.

Variables	n	(%)
**Age**		
18–20	141	(35.3)
21–23	124	(31.0)
24–26	135	(33.8)
**Study year**		
1st	78	(19.5)
2nd	94	(23.5)
3rd	87	(21.8)
4th	84	(21.0)
Master’s	57	(14.2)
**Marital status**		
Unmarried	344	(86.0)
Married	56	(14.0)
**Family history of breast cancer**		
Yes	73	(18.3)
No	327	(81.8)
**Relationship with affected patient**		
Mother	8	(11.6)
Sister/cousin	17	(24.6)
Aunt	28	(40.6)
Grandmother	16	(23.2)

### Knowledge about breast cancer’s symptoms, risk, treatment, prevention, screening, and process of screening of breast cancer

The mean score of knowledge about breast cancer symptoms was 2.94 (SD = 1.14) out of 8, with an overall correct rate of 36.8% (Table 2). The most common symptoms that were answered correctly by participants were ‘color change of breast including redness or flaky skin’ (48.3%), ‘new lump in the breast or armpit’ (42.8%), ‘changes in breast shape and size including inverted nipple’ (40.3%), and ‘nipple discharge other than breast milk including blood or pus’ (29.8%). The mean score of knowledge about breast cancer symptoms was significantly higher among participants of fifth year (Master’s). The mean score of knowledge about breast cancer risk was 3.35 (SD = 1.19) out of 10, with an overall correct rate of 33.5%. The mean score of knowledge about breast cancer risk was significantly higher among participants who reported being 24–26 years, Master’s students, and having other sources of information. The mean score of knowledge about breast cancer treatment was 1.80 (SD = 0.93) out of 6, with an overall correct rate of 30.0%. The mean score of knowledge about breast cancer treatment was significantly higher among participants who reported being 24–26 years and Master’s students.

**Table 2:** Distribution of participants’ knowledge about breast cancer’s symptoms, risk, treatment, prevention, screening, and process of breast self-examination.

Variables	Symptoms			Risk			Treatment			Prevention			Screening			Process of BSE		
	Total score = 8			Total score = 10			Total score = 6			Total score = 8			Total score = 5			Total score = 5		
	Mean (SD)	t/F	*p*-value	Mean (SD)	t/F	*p*-value	Mean (SD)	t/F	*p*-value	Mean (SD)	t/F	*p*-value	Mean (SD)	t/F	*p*-value	Mean (SD)	t/F	*p*-value
**Age**																		
18–20	2.82 (1.29)	1.81	0.166	3.03 (1.16)	8.37	**<0.001**	1.89 (1.02)	4.77	**0.009**	3.34 (1.11)	1.23	0.294	1.76 (0.54)	1.42	0.243	1.56 (1.9)	0.26	0.772
21–23	3.09 (1.04)			3.48 (1.23)			1.59 (0.96)			3.13 (1.12)			1.75 (0.47)			1.48 (1.8)		
24–26	2.93 (1.05)			3.56 (1.12)			1.9 (0.75)			3.3 (1.2)			1.84 (0.61)			1.64 (1.89)		
**Study year**																		
First year	2.55 (1.33)	6.40	**<0.001**	3.22 (1.12)	5.84	**0.016**	1.86 (0.81)	4.50	**0.001**	3.14 (1.2)	2.65	**0.033**	1.86 (0.55)	0.66	0.623	1.79 (1.98)	0.82	0.516
Second year	2.83 (1.08)			2.98 (1.24)			1.62 (1.05)			3.03 (1.14)			1.79 (0.53)			1.52 (1.89)		
Third year	2.98 (1.04)			3.26 (1.19)			1.63 (0.88)			3.3 (1.06)			1.78 (0.49)			1.55 (1.74)		
Fourth year	2.93 (1.07)			3.61 (0.98)			1.82 (0.78)			3.26 (1.23)			1.77 (0.6)			1.32 (1.74)		
Fifth year (Master’s)	3.4 (1.01)			3.68 (1.27)			2.14 (0.96)			3.58 (1.05)			1.88 (0.59)			1.85 (2)		
**Marital status**																		
Unmarried	2.92 (1.16)	1.36	0.245	3.36 (1.18)	0.19	0.664	1.82 (0.94)	1.48	0.225	3.28 (1.14)	1.16	0.282	1.83 (0.54)	2.32	0.128	1.58 (1.84)	0.06	0.813
Married	3.11 (1.04)			3.29 (1.25)			1.66 (0.79)			3.11 (1.19)			1.71 (0.56)			1.52 (1.98)		
**Family member of breast cancer**																		
Yes	3.08 (1.2)	1.34	0.248	3.48 (1.24)	1.05	0.305	1.88 (0.96)	0.61	0.434	3.18 (1.22)	0.46	0.500	1.73 (0.65)	2.50	0.115	4.25 (1.7)	341.87	**<0.001**
No	2.91 (1.13)			3.32 (1.18)			1.78 (0.92)			3.28 (1.13)			1.84 (0.52)			0.98 (1.28)		
**Source of information**																		
Friends/relatives	2.92 (1.21)	1.20	0.311	3.09 (1.27)	4.40	**0.002**	1.77 (1.04)	0.20	0.941	3.19 (1.2)	1.46	0.214	1.77 (0.55)	0.63	0.644	1.84 (1.99)	1.64	0.163
Social media	2.86 (1.2)			3.08 (1.19)			1.8 (0.89)			3.38 (1.25)			1.81 (0.53)			1.27 (1.74)		
Health campaign	2.84 (1.1)			3.55 (1.13)			1.86 (0.92)			3.22 (1.08)			1.86 (0.6)			1.65 (1.93)		
Doctors	3.18 (1.03)			3.47 (1.22)			1.74 (0.9)			3.47 (1.2)			1.87 (0.46)			1.27 (1.57)		
Others	3.09 (1.1)			3.73 (0.91)			1.8 (0.79)			3 (0.85)			1.76 (0.57)			1.78 (1.94)		
**Practice of BSE**																		
Yes	2.96 (1.16)	0.04	0.840	3.29 (1.29)	0.24	.627	1.85 (0.93)	0.28	0.598	3.13 (1.26)	1.41	.236	1.82 (0.6)	0.01	0.909	4.98 (0.15)	3792.90	**<0.001**
No	2.94 (1.14)			3.37 (1.17)			1.79 (0.93)			3.3 (1.11)			1.82 (0.53)			0.65 (0.64)		

The mean score of knowledge about breast cancer prevention was 3.26 (SD = 1.14) out of 8, with an overall correct rate of 36.2%. The mean score of knowledge about breast cancer prevention was significantly higher among participants who reported being Master’s students. The mean score of knowledge about breast cancer screening was 1.82 (SD = 0.55) out of 5, with an overall correct rate of 36.4%. The mean score of knowledge about breast cancer screening was not significantly different among participants in terms of socio-demographic groups or source of information. The mean score of knowledge about BSE process was 1.57 (SD = 1.86) out of 5, with an overall correct rate of 31.4%. The mean score of knowledge about BSE process was significantly higher among participants who reported having family members of breast cancer and those who have practiced BSE in the past.

**Fig 1:**
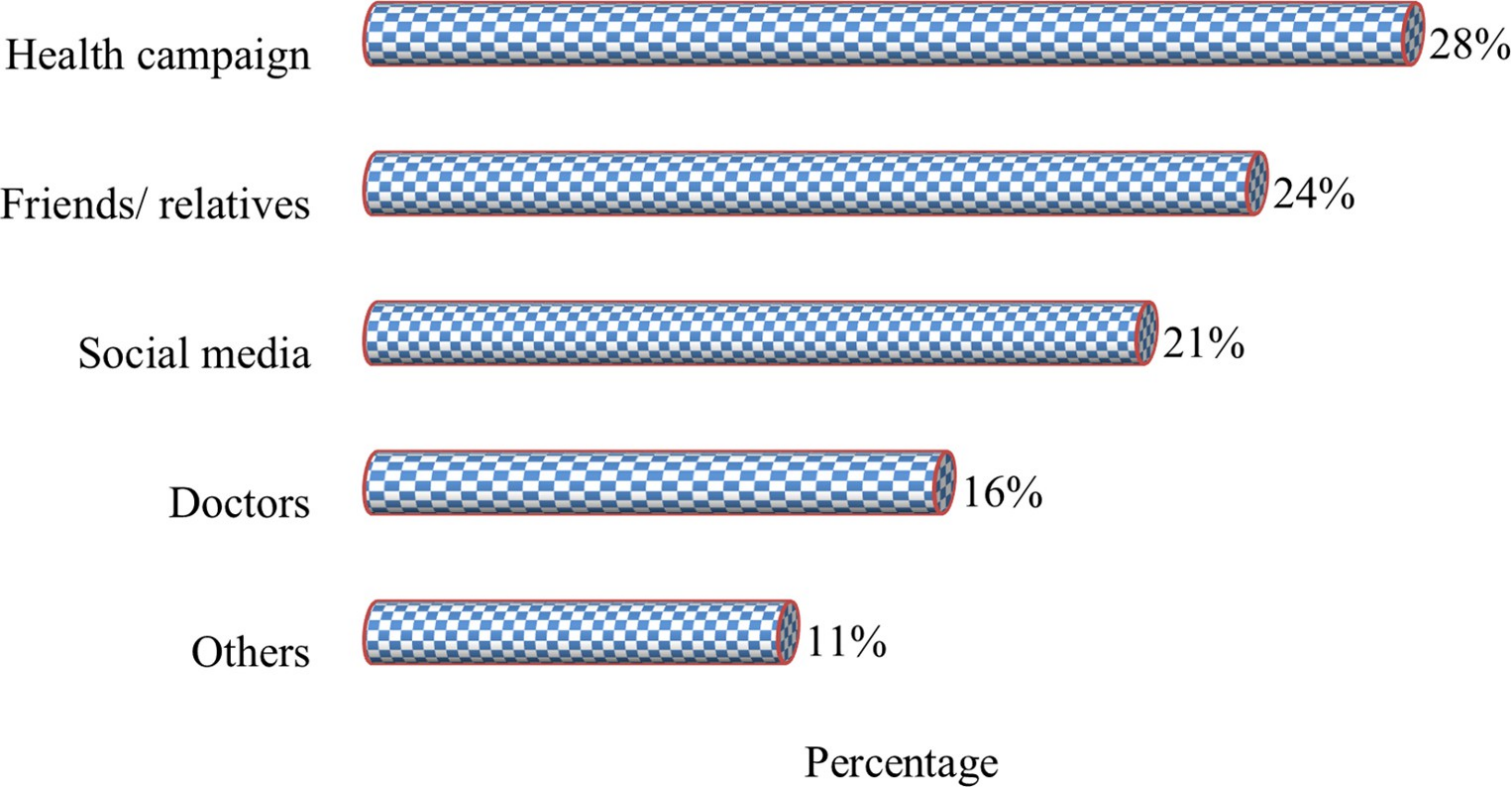
Source of information about breast cancer.

Overall, the mean score of total knowledge items was 14.74 (SD = 3.15) out of 43, with an overall correct rate of 34.3% (Table 3). The mean score of total knowledge items was higher among Master’s students and having family members with breast cancer. The participants’ sources of information about breast cancer are presented in Fig 1.

**Table 3:** Participants’ total knowledge and practice.

	Knowledge			Practice		
	Mean (SD)	t/F	*p*-value	Mean (SD)	t/F	*p*-value
**Age**						
18–20	14.54 (3.25)	1.83	0.162	0.22 (0.42)	0.19	0.825
21–23	14.52 (3.16)			0.19 (0.4)		
24–26	15.16 (3.01)			0.22 (0.42)		
**Study year**						
First year	15.14 (3.19)	3.47	**0.008**	0.26 (0.44)	0.81	0.520
Second year	13.77 (3.34)			0.21 (0.41)		
Third year	14.77 (3.01)			0.18 (0.39)		
Fourth year	14.82 (2.79)			0.17 (0.37)		
Fifth year (Master’s)	15.34 (3.12)			0.26 (0.44)		
**Marital status**						
Unmarried	14.8 (3.2)	0.80	0.371	0.21 (0.41)	0.15	0.699
Married	14.39 (2.81)			0.23 (0.43)		
**Family member of breast cancer**						
Yes	17.59 (2.5)	89.24	**<0.001**	0.84 (0.37)	427.67	**<0.001**
No	14.11 (2.92)			0.07 (0.26)		
**Source of information**						
Friends/relatives	14.58 (3.27)	1.13	0.341	0.27 (0.45)	1.52	0.195
Social media	14.2 (3.02)			0.17 (0.37)		
Health campaign	14.97 (3.53)			0.23 (0.42)		
Doctors	15 (2.37)			0.13 (0.34)		
Others	15.16 (3.01)			0.24 (0.43)		

### Practices of breast self-examination

About one-fifth of participants reported they had ever practiced BSE (21.3%). The mean score of practice of BSE was significantly higher among participants who reported having family members of breast cancer. Descriptive statistics and correlations between all outcome variables (i.e., knowledge about breast cancer’s symptoms, risk, treatment, prevention, screening, and process of breast self-examination, practice of breast self-examination) are presented in Table 4. It is noted that the total knowledge score about breast cancer and practice of BSE are significantly correlated with each other (*r* = 0.54; *p*<0.001).

**Table 4:** Descriptive statistics, and correlations between all outcome variables.

Variables	Kurtosis (SE)	Skewness (SE)	Mean (SD)	Range	1	2	3	4	5	6	7
1. Symptoms	0.06 (0.24)	-0.13 (0.12)	2.94 (1.14)	0–8	⸻						
2. Risk	-0.42 (0.24)	-0.2 (0.12)	3.35 (1.19)	0–10	.153**	⸻					
3. Treatment	-0.13 (0.24)	0.27 (0.12)	1.8 (0.93)	0–6	-.039	.286**	⸻				
4. Prevention	-0.46 (0.24)	0.00 (0.12)	3.26 (1.14)	0–9	.002	.040	.011	⸻			
5. Screening	0.62 (0.24)	-0.08 (0.12)	1.82 (0.55)	0–5	.047	-.009	.066	-.028	⸻		
6. Process of BSE	-0.4 (0.24)	1.1 (0.12)	1.57 (1.86)	0–5	-.003	.025	.048	-.035	-.023	⸻	
7. Overall knowledge	-0.15 (0.24)	0.2 (0.12)	14.74 (3.15)	0–43	.416**	.546**	.432**	.357**	.184**	.597**	⸻
8. Practice	-0.01 (0.24)	1.41 (0.12)	0.21 (0.41)	0–1	.010	-.024	.026	-.059	.006	.951**	.544**

Note:

SE = standard error; SD = standard deviation.

**Correlation is significant at the 0.01 level (2-tailed).

### Barriers of breast self-examination

About 33.3% of the participants have addressed ‘lack of knowledge’ as the main barrier to practicing BSE followed by ‘I do not have the symptoms’ (21.8%), ‘shyness/ uncomfortable feelings’ (16.5%), ‘I don’t think it’s important (9.5%), ‘I know I will never have breast cancer’ (6.8%), and ‘fear or being diagnosed of cancer’ (6.3%).

## Discussion

The present study explored knowledge of breast cancer and practice of BSE among female university students in Bangladesh. The present findings on the common symptoms are in consensus with a study conducted in Saudi Arabia which also showed a moderate knowledge of ‘presence of lump in the breast’ (48%) and ‘change in the shape of the breast or nipple’ (42.9%), although ‘inverted nipple’ was known as a warning sign of breast cancer by only 29% of the students [16]. In another study of female university students in Egypt, breast lump was the most commonly identified (81.6%) symptom of breast cancer [17], though the percentage was significantly higher than our study implying that the awareness regarding symptoms of breast cancer needs to be improved in Bangladesh.

Our findings on levels of knowledge about risk factors of breast cancer were also consistent with the Saudi Arabian study, which also showed that only 42.3% of the study sample knew that lack of physical exercise increases the risk of breast cancer [16]. Consistent with our study, a number of studies with different groups of women in Malaysia have also found that a lack of knowledge of breast cancer was the most commonly identified addressed barrier to breast cancer prevention practices [20, 21]. A moderate number of respondents in our study had positive attitudes toward the early detection of breast cancer, which has also been found in Iraq [22]. In the study conducted in Iraq, the participants had low knowledge on the treatment options for breast cancer—only 40.3% stated chemotherapy, 37.3% stated surgery and 27.8% stated hormonal therapy. In comparison, a study conducted with female university students in Egypt found a significantly higher percentage of students recognized chemotherapy and surgery as treatment options [17], suggesting that students in Bangladesh may require further information on treatment options for breast cancer so they know it can be treated and thus are aware of the reasons and benefits for BSE. Interestingly, respondents in our study believed that alternative medicines (17.0%) and herbal treatments (20.5%) are effective treatment options for breast cancer. We need to keep in mind that our sample was women currently at university who one may assume have access to the most up-to-date information sources and are thus among the most literate, health literate and digitally literate women in Bangladesh. Therefore, if levels of knowledge are low in this group, we assume they would be even lower in other groups of women who do not go to university. As such, evidence-based educational materials may be required to provide women (at different literacy levels and therefore in different ways) in Bangladesh for effective treatments for breast cancer.

BSE was known to only 42.0% of the respondents in our study, a finding consistent with a study in Pakistan which found that only 40.3% of students knew about BSE [23]. CBE was the least known method for breast screening in our study, whereas 64.2% had heard of CBE in a different study conducted among women aged 30–59 years was conducted in Bangladesh [24]. Also, in contrast with our study, a study with female university students in Egypt found that 74.2% know about BSE, 52.1% about mammogram and 48.3% about ultrasound [17], which is considerably higher than in our study. In our study, although 42.0% knew about BSE, only 21.3% of the respondents had ever practiced BSE, which is similar to another study in Bangladesh whereby 46.7% of respondents knew about BSE but only 16.3% had ever practiced BSE [25]. Indeed, the practice rate of BSE by female students is low in a number of countries, such as Cameroon (38.5%), Yemen (17.4%), Iraq (19.7%), Turkey (20.3%), Egypt (6.1%), and Korea (27%) [17, 19, 22, 26–28].

In our study, there was a significant relationship between the overall knowledge score and practice of BSE, implying that the more knowledgeable participants are also more likely to practice breast self-examination. We are acutely aware that correlation does not equal causation, but it seems plausible that knowledge of breast cancer would impact BSE as opposed to the other way around. This supports the fact that knowledge about breast cancer can play a vital role in the increased breast awareness and the practice of BSE. As Bangladesh is a developing country with low resources, mammography as a screening procedure is not a feasible first-line prevention or detection procedure. Therefore, BSE and CBE should be the main screening procedure for breast cancer in Bangladesh. Television/radio/mass media were identified as the main source of information in our study and this was similar in other studies [17, 29, 30], which highlights the key mechanisms for informing women about breast cancer risks, prevention and treatment, and also the importance of BSE.

The number of years that our participants had been studying had a significant association (*p* < .001) with breast cancer knowledge about symptoms, risk factors and treatment options, indicating that higher the educational level is associated with higher knowledge about breast cancer. In addition, those who had a family history of breast cancer with close relatives had significantly higher knowledge about BSE procedure (*p* < .001). A similar association was also found in Saudi Arabia [31].

A Malaysian study found that knowledge of breast cancer was low among young and less educated women [32]. On the contrary, a study conducted in India found no significant association found between demographic variables and level of knowledge of breast cancer and BSE among women [33] and younger women in college [34]. These indicate that in different populations, the association level may vary on the basis of social, cultural and demographic factors. The present study indicated that the knowledge gap was the main driving factor that impeded BSE practices among participants, although we only included women enrolled at university so we cannot comment on educational and socio-economic differences with other women in Bangladesh.

### Limitations

All the data were self-reported by the participants and there is chance of recall bias. No verification could be undertaken to validate participants’ claims to practice (or not) BSE. Also, the accuracy, timing, frequency and interpretation of BSE practice were not assessed. Moreover, we conducted the study on female students of only one university using cross-sectional methods. Given that our sample are highly educated, we also cannot claim any generalizability to other groups of women in Bangladesh. So, a future study with larger sample sizes including a broader cross-section of the population (e.g., high school students, women in poverty, women in different socio-economic groups, women working in a particular sector) and prospective methods is warranted to overcome the potential study limitations.

### Conclusions

The present study reveals that university female students had limited knowledge on breast cancer, ranging from 30% about treatment options to 37% about symptoms, with overall correct rate of knowledge being 34.3%. Moreover, the present study revealed that the practice of BSE was low in our sample. This study also found a significant association between knowledge of breast cancer and practice of BSE, implying appropriate knowledge and awareness about breast cancer may lead to practice of BSE and thus early diagnosis which in terms can help to reduce the breast cancer morbidity and mortality. More studies need to be conducted to find out the baseline knowledge and practice about breast cancer across the general population and between different socio-demographic groups so that effective preventive policies can be developed and implemented. Also, educational interventions programs that are socially, demographically and culturally appropriate should be promoted and implemented to raise awareness regarding breast cancer and enhance BSE practice among all females in Bangladesh.

## Supporting information

S1 File Questionnaire. (DOCX)

S2 File Distributions (frequencies and percentages) of all knowledge related questions. (DOCX)

S3 File Data set. (XLSX)
